# Using Colonization Assays and Comparative Genomics To Discover Symbiosis Behaviors and Factors in Vibrio fischeri

**DOI:** 10.1128/mBio.03407-19

**Published:** 2020-03-03

**Authors:** Clotilde Bongrand, Silvia Moriano-Gutierrez, Philip Arevalo, Margaret McFall-Ngai, Karen L. Visick, Martin Polz, Edward G. Ruby

**Affiliations:** a University of Hawaii—Manoa, Honolulu, Hawaii, USA; b Department of Civil and Environmental Engineering, Massachusetts Institute of Technology, Cambridge, Massachusetts, USA; c Department of Microbiology and Immunology, Loyola University Chicago, Maywood, Illinois, USA; University of Connecticut

**Keywords:** *Vibrio* (*Aliivibrio*) *fischeri*, genomes, dominance, symbiosis, intraspecific, *Aliivibrio*, *Vibrio fischeri*, genome analysis

## Abstract

The luminous marine Gram-negative bacterium *Vibrio* (*Aliivibrio*) *fischeri* is the natural light organ symbiont of several squid species, including the Hawaiian bobtail squid, Euprymna scolopes, and the Japanese bobtail squid, Euprymna morsei. Work with E. scolopes has shown how the bacteria establish their niche in the light organ of the newly hatched host. Two types of V. fischeri strains have been distinguished based upon their behavior in cocolonization competition assays in juvenile E. scolopes, i.e., (i) niche-sharing or (ii) niche-dominant behavior. This study aimed to determine whether these behaviors are observed with other V. fischeri strains or whether they are specific to those isolated from *E. scolopes* light organs. Cocolonization competition assays between V. fischeri strains isolated from the congeneric squid E. morsei or from other marine animals revealed the same sharing or dominant behaviors. In addition, whole-genome sequencing of these strains showed that the dominant behavior is polyphyletic and not associated with the presence or absence of a single gene or genes. Comparative genomics of 44 squid light organ isolates from around the globe led to the identification of symbiosis-specific candidates in the genomes of these strains. Colonization assays using genetic derivatives with deletions of these candidates established the importance of two such genes in colonization. This study has allowed us to expand the concept of distinct colonization behaviors to strains isolated from a number of squid and fish hosts.

## Abstract (precis)

There is an increasing recognition of the importance of strain differences in the ecology of a symbiotic bacterial species and, in particular, how these differences underlie crucial interactions with their host. Nevertheless, little is known about the genetic bases for these differences, how they manifest themselves in specific behaviors, and their distribution among symbionts of different host species. In this study, we sequenced the genomes of Vibrio fischeri isolated from the tissues of squids and fishes and applied comparative genomics approaches to look for patterns between symbiont lineages and host colonization behavior. In addition, we identified the only two genes that were exclusively present in all V. fischeri strains isolated from the light organs of sepiolid squid species. Mutational studies of these genes indicated that they both played a role in colonization of the squid light organ, emphasizing the value of applying a comparative genomics approach in the study of symbioses.

## INTRODUCTION

*Vibrio* (*Aliivibrio*) *fischeri* is the light-emitting, bacterial symbiont of several fish and squid species (1). These bacteria become established within the host animal’s light organ and provide bioluminescence in exchange for nutrients. Strains of Vibrio fischeri have been isolated from the light organs of the squids Euprymna scolopes, Euprymna morsei, and Euprymna tasmanica, the light organs of the fishes Monocentris japonica and Coelorinchus multispinulosus, and from the accessory nidamental gland (ANG) of the squid Loligo pealei. The ANG is a female-specific structure that protects the squid’s eggs from biofouling by covering them with a bacterial community producing antifungal compounds (2, 3).

When juvenile E. scolopes hatch, they harvest V. fischeri cells from the surrounding environment using ciliated fields present on surface appendages of the light organ (4). These bacteria then pass through pores on the organ’s surface and establish themselves in the deep crypts of the light organ (5, 6). Each dawn, almost all of the light organ’s symbiont population is expelled from the crypts into the surrounding seawater: the remaining bacteria multiply and repopulate the organ, while those released into the ambient environment serve as an inoculum for juvenile squid that hatch on subsequent nights (6). Phenotypic differences among symbiotic V. fischeri strains found around the island of Oahu, Hawaii, have been reported (7–10). In particular, two behaviors have been described when juvenile squid were coinoculated with a pair of strains: a niche-dominant (D) behavior, where only one of the strains establishes in the squid light organ, and a niche-sharing (S) behavior where coinoculated strains both inhabit the organ (9). A type VI secretion system (T6SS) has recently been identified in some strains of V. fischeri, and carriage of this system can lead to interstrain killing within cocolonized light organ crypts (11). However, because not all D-type strains carry the lethal system and some S-type strains do, there is no evidence from comparative genomics that T6SS activity is responsible for the D behavior. Instead, we recently showed that D strains reach, and begin growing within, the crypts earlier than S strains, a competitive advantage that makes it difficult for S strains to secondarily colonize a light organ (12). One might expect that this colonization advantage would allow D strains to ecologically “sweep” a symbiont population, but several factors work against that outcome. (i) The evolution of V. fischeri is unlikely to be governed solely by symbiotic behavior because only a vanishingly small percentage of the billions of symbionts released into the bacterioplankton each day ever encounter a host again (13). (ii) The D strains appear to be less fit at surviving in the bacterioplankton than the S strains (7). (iii) If the newly hatched squid encounters an S strain sufficiently long before a D strain, both strains can establish in the light organ (12).

**FIG 1:**
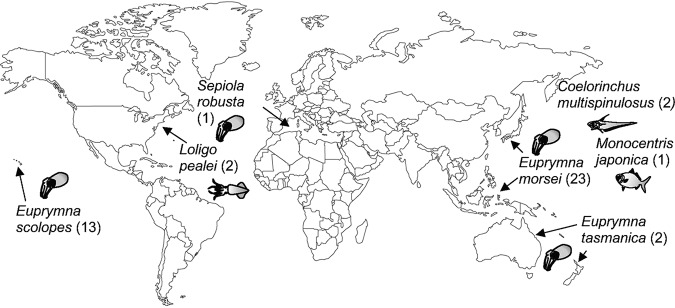
Locations and species from which Vibrio fischeri strains involved in this study were isolated. The number of isolates from each host species is shown in parentheses in the figure. Twenty-nine of these strains (the *E. morsei*, *E. tasmanica*, *L. pealei*, and *C. multispinulosus* isolates) were sequenced in this study.

In this study, we aimed to determine whether the dominant and sharing behaviors described for *E. scolopes* light organ symbionts were also observed with 31 additional V. fischeri strains isolated from other hosts, tissues, and geographic regions (Fig. 1) by performing coinoculation assays using juvenile *E. scolopes* as the host. We also compared the genomes of all of these V. fischeri strains to determine whether there was a correlation between colonization behavior and either genome relatedness or gene content. In addition, we sought to identify genetic determinants specific to strains defined by their behavior or by their host. As a result of this work, we showed the following. (i) D and S behaviors are a common feature within V. fischeri strains isolated from different hosts. (ii) While some dominant strains clustered together on a phylogenetic tree, there is no obvious pattern or genetic determinant that explains the behavior of all dominant strains. (iii) Comparative genomics revealed two new genes, encoding a putative enoyl-coenzyme A (CoA) hydratase and a LysR-family transcriptional regulator, found only in squid light organ isolates, that play a role in symbiotic colonization.

## RESULTS AND DISCUSSION

### Niche-dominant (D) or -sharing (S) behaviors are observed among all V. fischeri strains tested, independent of their host origin.

**FIG 2:**
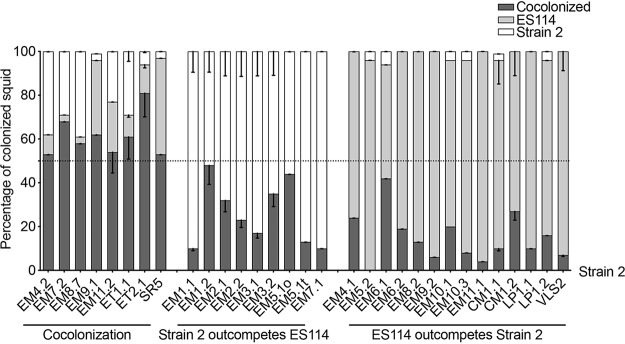
State of colonization of juvenile *E. scolopes* squid coinoculated with two strains. Squid were inoculated for 3 h with strain ES114 and a second strain of V. fischeri (“Strain 2”; strains listed across the *x* axis), and the percentages of squid that were cocolonized, colonized only by strain ES114, or colonized only by strain 2 were determined after 24 h. Each bar represents data collected from between 26 and 34 animals, analyzed in three replicates. The graph indicates the mean percentages for each result, and the error bars give the 95% confidence intervals. Strains were isolated from the light organs of the fish *C. multispinulosus* (CM), the squid accessory nidamental gland of *L. pealei* (LP), or the squid light organs of *E. morsei* (EM), *E. scolopes* (ES) (VLS2), *E. tasmanica* (ET), or *S. robusta* (SR).

Our first aim was to determine whether D or S behavior was observed when juvenile *E. scolopes* were coinoculated with strains isolated from other hosts. Coinoculation with the well-studied V. fischeri S strain ES114, isolated from *E. scolopes* (14), was chosen as a means to identify sharing behavior in other strains because of its low position on the dominance-hierarchy scale (9). Thus, juvenile squid were coinoculated with strain ES114 and 1 of 30 other strains for 3 h, and the relative numbers of these 2 strains were determined after 48 h (Fig. 2). Three outcomes were identified. (i) A strain outcompeted strain ES114, resulting in a mostly single-strain colonization of the light organ, and was considered a D strain. (ii) A strain cocolonized >50% of the squid with ES114 and was considered an S strain. (iii) A strain was outcompeted by ES114, typically resulting in a single-strain colonization of the squid by ES114. We determined that, among the isolates tested, each of these three behaviors was found in approximately the same numbers of strains tested: 8, 9, and 14, respectively.

**FIG 3:**
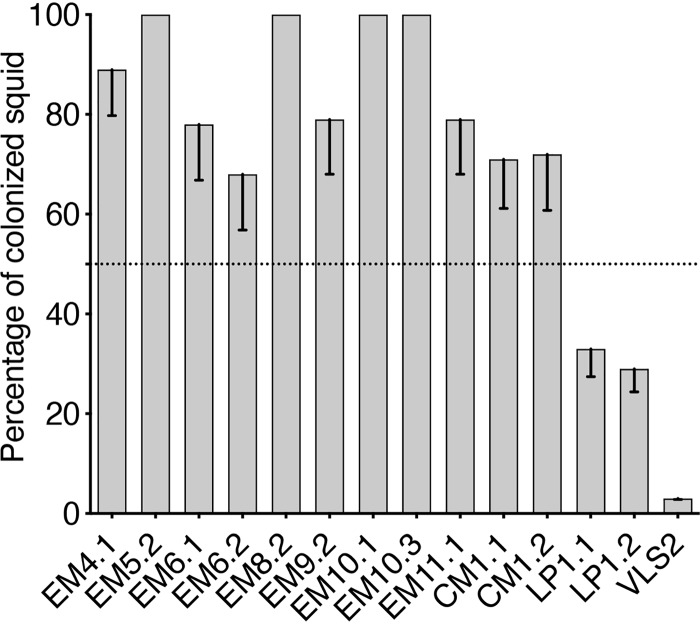
Colonization success of squid that were inoculated with a single V. fischeri strain. Squids were inoculated for 3 h with an individual strain from the 14 strains outcompeted by strain ES114 in Fig. 2 (right). Each bar represents the percentage of colonized squid based on their bioluminescence at 24 h postinoculation. For each condition, between 30 and 41 animals were analyzed in three replicates. The graph indicates the mean percentages for each result, and the error bars give the 95% confidence intervals.

We realized that the class that was outcompeted by strain ES114 might include certain V. fischeri strains that are simply unable to colonize *E. scolopes* under ecologically relevant conditions, as previously reported for the *Monocentris japonica* isolate strain MJ11 (15). To identify such symbiosis incompetence, groups of squid were inoculated solely with the strain in question, and the number of colonized animals was determined after 24 h by individually measuring their bioluminescence. With the exception of strains LP1.1, LP1.2, and VLS2, all the strains were capable of initiating symbiosis in most of the juvenile squid when they were the only V. fischeri present (Fig. 3). Interestingly, strains that could colonize had been isolated from symbiotic light organs, while two of the poor colonizers, LP1.1 and LP1.2, were obtained from a loliginid squid’s ANG. The symbiotic light organs of loliginids have been reported to be colonized by the bioluminescent species Photobacterium leiognathi and perhaps Vibrio harveyi (16), and V. fischeri strains have yet to be reported in the ANG of sepiolid squids (17). The poor colonization ability of VLS2, an *E. scolopes* light organ isolate (unpublished data), is unique among all other squid light organ isolates reported, and the basis of its incompetence is unknown.

**FIG 4:**
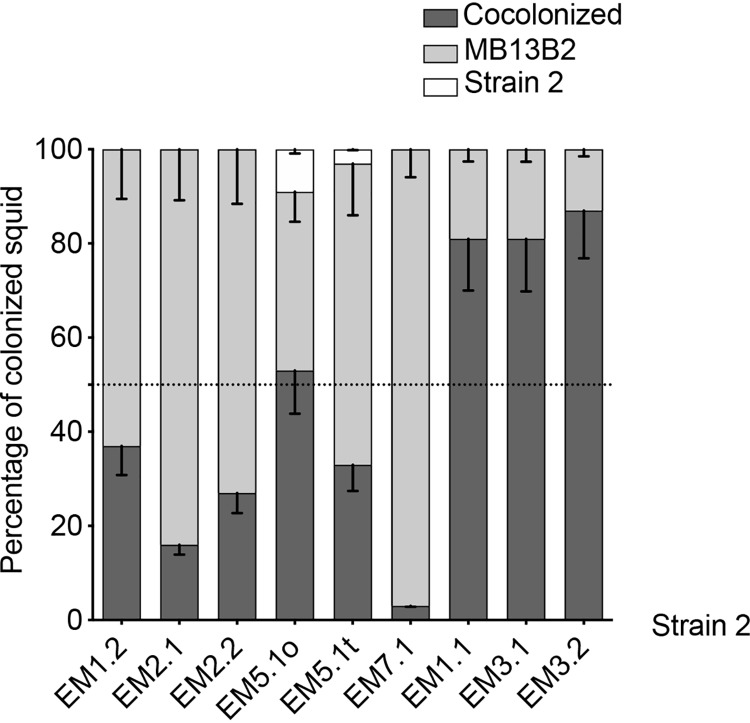
State of colonization of coinoculated juvenile *E. scolopes* squid. Squids were inoculated for 3 h with both the dominant (D) strain MB13B2 and a second strain (“Strain 2”; listed across the *x* axis) that were the 9 most dominant strains in Fig. 2 (middle). The percentages of squid that were cocolonized , colonized only by strain MB13B2 or only by Strain 2 were determined after 24 h. For each condition, between 30 and 32 animals were analyzed in three replicates. The graph indicates the mean percentages for each result, and the error bars give the 95% confidence intervals.

We next assessed the behavior of strains that outcompeted strain ES114 (i.e., had a D-type phenotype) by competing them against one of the more dominant *E. scolopes* D strains, MB13B2 (9). As expected for D strains, a single strain was present after cocolonization with most of the strain pairings (Fig. 4). In these cases, MB13B2, a strain isolated from an *E. scolopes* light organ, was generally dominant over the strains isolated from other animals. In contrast, when squid were coinoculated with MB13B2 and either EM1.1, EM3.1, or EM3.2, most of the light organs were colonized by both strains, suggesting that even symbionts from a different host species can robustly colonize a nonnative host.

Using the cocolonization assay, we described four classes of strain behavior, in order of their effectiveness: (i) D strains, (ii) S strains, (iii) strains outcompeted by an S strain, and (iv) ineffective colonizers. Of the 44 strains tested, only 4 were unable to colonize more than 50% of the squid under our laboratory inoculation conditions. By increasing the inoculum and/or the time of exposure, these strains could colonize a few squid; however, it seems less likely that they would become symbiotic in the natural environment if more competitive strains are present. The third class is a new category: strains that can colonize when they are on their own but are outcompeted by the S strain ES114. None of these strains were isolated from *E. scolopes*, so perhaps these strains have a disadvantage when encountering a nonnative host, although there is no evidence of coevolution between different sepiolid squid host species and their symbiont strains (1, 9). Alternatively, this result may indicate that it is more appropriate to refer to D and S behaviors, rather than D and S strains, because these behaviors are dependent on the pairing of strains being presented to the host. Similarly, it should be noted that these D and S behaviors occurred during colonization of juvenile *E. scolopes*; the outcome might be different in the native host of the strain (18). However, we have performed coinoculation experiments with two genetically distinct populations of *E. scolopes* and observed no effect of genotype (9); if this is an indication of the effect of host genetics in general on light organ symbionts, it is possible that the host species will have little effect (and, thus, the D and S behaviors may be more a symbiont characteristic than a host selection trait [12]). Not surprisingly, both environmental and intrahost selection mechanisms have been reported to play a role in other symbioses (19).

**FIG 5:**
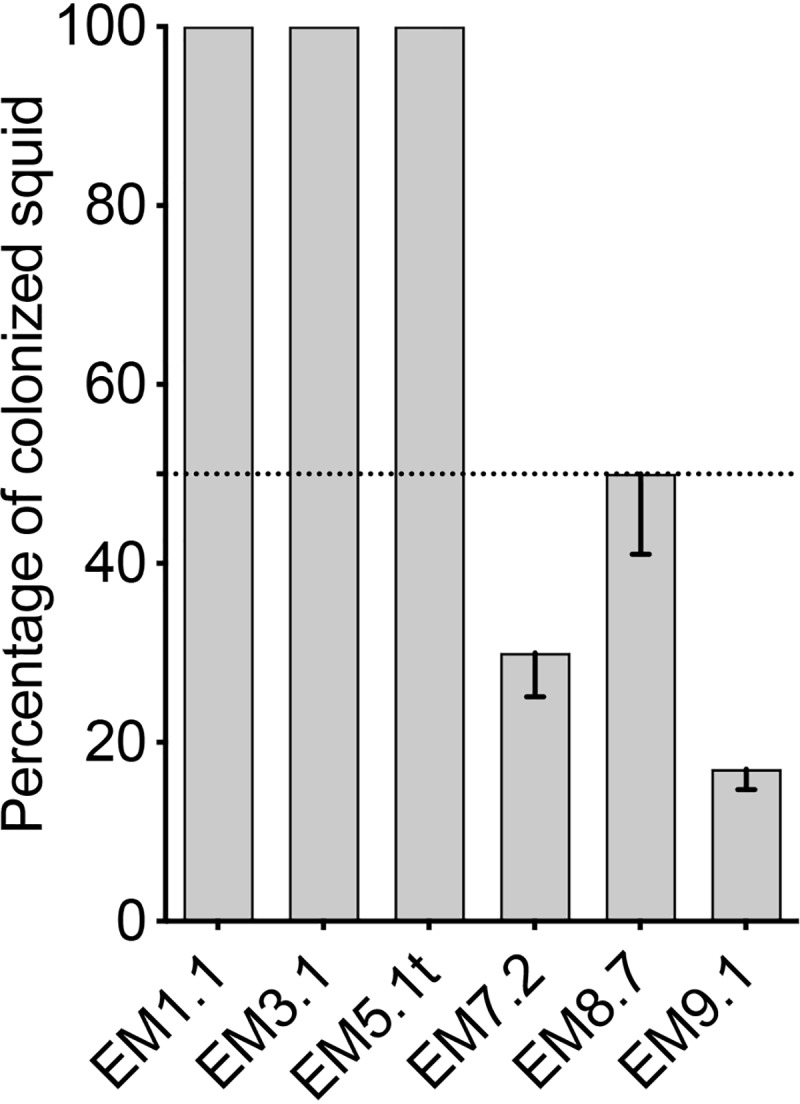
Colonization success of squid that were inoculated with a single V. fischeri strain for 30 min and rinsed three times. The V. fischeri strains are shown on the *x* axis. Each bar represents the percentage of colonized squid, based on their bioluminescence at 24 h postinoculation. Between 29 and 30 animals were analyzed for each of the strains in three replicates. The graph indicates the mean percentages for each result, and the error bars give the 95% confidence intervals.

One way to differentiate between D and S behaviors is by the much shorter exposure time that D strains need to initiate a successful colonization (12). Therefore, we exposed juvenile squid to pairs of three newly identified S and D strains for only 30 min, rinsed the squid to remove the inoculum, and checked the host for bioluminescence after 24 h (Fig. 5). As expected, the three presumptive D strains had a 100% colonization success, while the S strains colonized less than 50% of the squid; thus, this assay for colonization effectiveness is both valid and predictive.

### Linking genomes and colonization behaviors.

In this study, we sequenced the genomes of 29 previously isolated strains of V. fischeri (see Table S1 in the supplemental material) (1, 20, 21) and compared them with the published genomes of 15 other natural isolates (9, 22). The total genome sizes of these 44 strains fell between 3.6 and 4.5 Mb, and all had a GC content between 38.0 and 39.1% (Table S2). In addition, while the core genome previously described for 12 *E. scolopes* isolates and the divergent monocentrid fish light organ isolate MJ11 consists of 3,170 orthologous proteins (9), adding the 29 new genomes from around the world (Fig. 1) reduced this number by only 3%. Thus, V. fischeri seems to have a relatively stable genome: specifically, this species has an unusually low mutation rate (23) and a genome size that remains within a relatively narrow range of 4.0 Mb ± 10%, which is considerably more restricted than the range seen for other reported *Vibrio* species (24–27). This high degree of similarity suggests that the pangenome of V. fischeri is relatively small, even among strains collected from around the world, and across time and host species.

10.1128/mBio.03407-19.1 TABLE S1 Strains described in this study. Download Table S1, PDF file, 0.04 MB. Copyright © 2020 Bongrand et al. 2020 Bongrand et al. This content is distributed under the terms of the Creative Commons Attribution 4.0 International license.

10.1128/mBio.03407-19.2 TABLE S2 Primers used in this study. Download Table S2, PDF file, 0.01 MB. Copyright © 2020 Bongrand et al. 2020 Bongrand et al. This content is distributed under the terms of the Creative Commons Attribution 4.0 International license.

**FIG 6:**
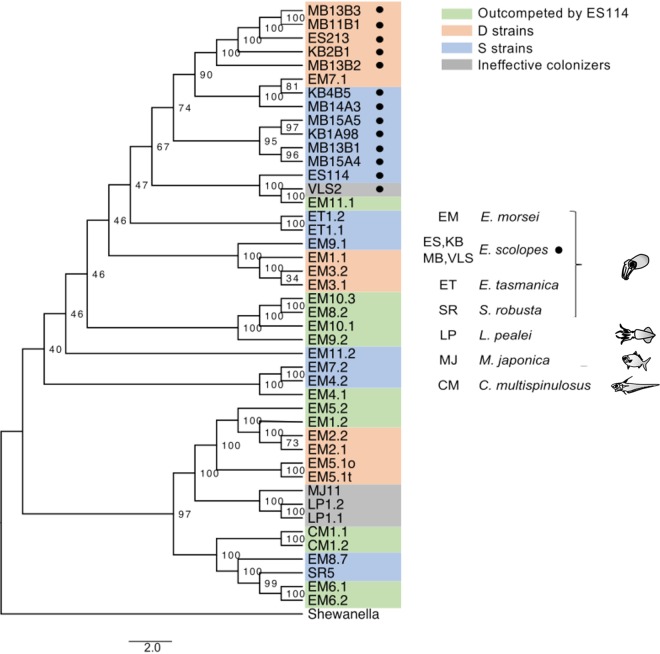
Phylogenetic relationships between the V. fischeri strains reported either in this study or in the study of Bongrand et al. (9), based on core genes from whole-genome sequences. Shewanella (Shewanella oneidensis) served as the outgroup. The scale bar indicates an evolutionary distance of 2 nucleotide substitutions per position in the sequence. Node numbers indicate the bootstrap support values. Most of the symbionts carry the initials of their host in their name. Because designations of *E. scolopes* symbionts use several different initials, we added a black circle to help identify them. The color code refers to the colonization effectiveness of the strains. The strains were either D strains (red), S strains (blue), outcompeted by strain ES114 (green), or ineffective colonizers of *E. scolopes* juveniles (gray).

We also asked whether there was any evolutionary signature that correlated with a strain’s colonization behavior. Specifically, we examined the relative positioning of (i) D strains, (ii) S strains, (iii) strains outcompeted by strain ES114, and (iv) ineffective colonizers, on a phylogenetic tree (Fig. 6). Interestingly, while the number of strains was insufficient to perform a robust statistical analysis, we noticed that although some strains with the same behavior clustered, others were spread across the tree; i.e., there is no obvious relationship between genome-wide phylogeny and behavior. Nevertheless, at the tips of the phylogenetic tree, there is some evidence of clustering of symbionts having the same colonization behavior, as has been found for the phylogenetic clustering of the degree of virulence of strains of the fish pathogen Vibrio anguillarum (28). The latter work also suggested a geographic association between similar strains, which we did not see. Our observation that all of the *E. scolopes* isolates cluster as the most recently diverged clade on the phylogenetic tree (Fig. 6) is in agreement with the fact that this host species is endemic to the Hawaiian Islands, where it is believed to have arisen (29).

To determine whether any proteins were specific to a certain V. fischeri behavioral group, we used OrthoMCl (30) to compare the proteins encoded in the sequenced genomes. We found no protein exclusive to the strains with S-type behavior, to those outcompeted by strain ES114, or to ineffective colonizers, which is consistent with the notion that the poor colonization ability of the two latter categories would be more likely due to an absence/loss of function (or gene) than to a gain. In contrast, a previous search for proteins specific to five *E. scolopes* strains with D-type behavior revealed 194 proteins exclusive to them (9); however, the addition of the new D-strain genomes described here halved this number to 89 (Table 1). More interestingly, there is no protein specific to all the D strains. Thus, unlike the symbiosis factor RscS (15), the dominance behavior reported here does not correlate with carrying a particular V. fischeri gene; however, it is important to note that differential behavior may also be linked to allelic differences among strains bearing a shared gene that has spread and been selected for in a specific environment (31). While identifying such an allele from our data would be difficult, the approach has been successful in other host-microbe systems. For example, the bacterium Aeromonas veronii, after several passages through its zebrafish host, evolved and selected for a more host-adapted derivative that was more effective at tissue migration (19). Similarly, analyses of Vibrio splendidus strains have provided evidence for allelic selection in two core genome genes that determine the bacterium’s interaction with oyster larvae (32). Thus, one can envision two possible reasons for the dispersed distribution of dominant behavior seen in Fig. 6. (i) It resulted from recombination and selection for an allele responsible for the behavior (33), an idea that could be tested by the examination of single-nucleotide polymorphism (SNP)-level differences linked to a specific behavior. (ii) There are several strategies for achieving dominance, and the behavior has evolved convergently among different V. fischeri lineages (e.g., see reference 10). For example, they may adapt more quickly to host-derived signals and/or challenges encountered during colonization (34, 35). This second possibility could be tested phylogenetically with the addition of sufficient V. fischeri genomes to create a robust tree of strains with known behaviors, from which an estimate of the ancestral behavioral state could be inferred. While we are beginning to understand the basis of strain variability in V. fischeri colonization (10), much is still to be learned about these phenotypes and their consequences to symbiosis.

**TABLE 1:** OrthoMCL analysis for groups of V. fischeri strains

Category of *V. fischeri* strains	No. of strains	No. of orthologous proteins^*a*^	Subcategory by host species	No. of strains	No. of orthologous proteins
By behavior					
D strains	14	0			
			*E. morsei* D strains	9	0
			*E. scolopes* D strains	5	89
S strains	15	0			
Outcompeted by strain ES114	11	0			
Inefficient colonizers	4	0			
					
By source					
Squid light organ	39	2			
			*E. morsei*	23	0
			*E. scolopes*	13	0
			*E. tasmanica*	2	12
			*S. robusta*	1	4
Fish light organ	3	4			
			*C. multispinulosus*	2	96
			*M. japonicus*	1	4
					
Squid ANG^*b*^	2	82	*L. pealei*	2	82

a Orthologous proteins found in all strains within that group but absent in all other groups; generally, groups comprising more strains have few or no unique orthologous proteins.

b ANG, accessory nidamental gland.

**FIG 7:**
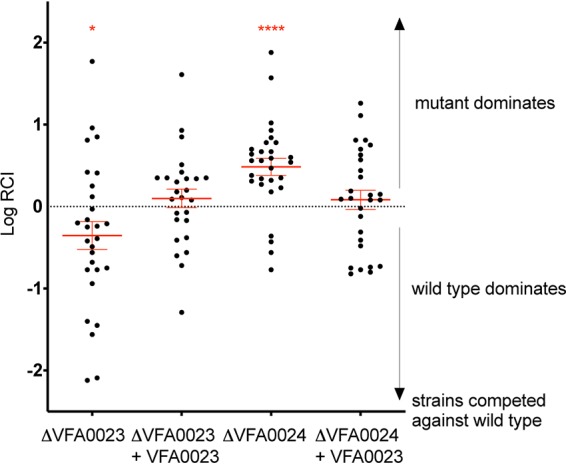
Relative competitive index of pairs of strains coincubated with juvenile squid for 3 h. The relative competitive index (RCI) was calculated as the ratio of the two strains in the light organ after 24 h, divided by their ratio in the inoculum. Those RCIs that are significantly different from zero by *t* test are indicated by red asterisks (*, *P* < 0.05; ****, *P* < 0.0001). The two strains competed were the parent ES114 strain and either a derived deletion mutant (Δ) or its genetic complement (+). Each dot represents the log RCI determined for an individual squid. For each condition, a total of between 26 and 29 animals were analyzed in three replicates. The error bars indicate the standard errors of the means (SEM).

We next asked whether a comparative genomic analysis could identify novel genes that were necessary not for a competitive behavior but for competence in light organ symbiosis. A previous comparison of the genomes of V. fischeri strains from *E. scolopes* had revealed 23 light organ-specific proteins (9). The present study, which added other V. fischeri genomes from light organ symbionts of other species of sepiolid squids, reduced this list of proteins to only two (Table 1). In strain ES114, the encoded proteins are named VF_A0023 and VF_A0024: the former is a putative enoyl-CoA hydratase, and the latter is a LysR-family transcriptional regulator. The two genes encoding these proteins are adjacent to each other and divergently transcribed in all 39 sepiolid squid symbionts. To determine whether these shared-derived proteins might play a role in establishing a light organ symbiosis, we cocolonized *E. scolopes* juveniles with the wild-type parent strain ES114 and each of the two single-gene mutant derivatives (Fig. 7). Because both of these mutants were present in the squid after coinoculation (and were also present in single colonization experiments), neither of these genes is required for colonization. However, the enoyl-CoA hydratase (VF_A0023) mutant was outcompeted by its parent, indicating a role for this enzyme in successfully initiating colonization. In contrast, the transcriptional regulator mutant (VF_A0024) showed the opposite phenotype, i.e., it outcompeted the wild-type ES114 during colonization, suggesting that this gene encodes a repressor of a positive colonization factor(s) (Fig. 7). Both mutant phenotypes were reversed by the presence of a plasmid encoding the deleted gene in the mutant strains. Removing the entire VF_A0023-0024 locus had no discernible effect on competitive fitness relative to the wild-type parent (data not shown), suggesting that these two genes can compensate for each other’s effects. Thus, a comparative genomic bioinformatic analysis has identified two new open reading frames (ORFs) involved in modulating squid colonization. Future studies will be required to determine the modes of action of these genes and their encoded activities.

In conclusion, we performed a comparative genomic and behavioral study of V. fischeri strains to identify genetic elements responsible for a colonization behavior. While no such genes were identified, we discovered two new genes required for the effective colonization of the squid light organ. Assessing the contribution of strain-level diversity in host colonization is a key element in understanding how a symbiont population is established. Studies of the squid-vibrio model allow us to examine strain diversity within a single species, and applying an analogous approach can also reveal mechanisms of colonization in more-complex communities. For example, the competitiveness of diverse bacterial species has been established in the bean bug Riptortus pedestris (36), while on a larger scale, the first bacterial population colonizing the mouse gut has been shown to most influence the composition of its final microbial community (37). The future application of comparative genomics will continue to provide a means to discover strain-level patterns in the evolution and development of microbial symbioses.

## MATERIALS AND METHODS

### Bacterial strains.

All Vibrio fischeri strains used in this study (see Table S1 in the supplemental material) were isolated from the tissues of squid or fish species (Fig. 1). Most of the strains were isolated from the light organs of one of four sepiolid squid species. Three of the strains were isolated from the light organs of two fish species: strain MJ11 from *Monocentris japonica* and strains CM1.1 and 1.2 from *Coelorinchus multispinulosus*. Strains LP1.1 and 1.2 came from the accessory nidamental gland (ANG) of the squid *Loligo pealei*. Strains sharing the same first numerical indicator were isolated from the same host (e.g., EM1.1 and EM1.2 or MB15A4 and MB15A5). To differentiate strains used in the cocolonization assays, we labeled ES114 and MB13B2 with a gene encoding a green fluorescent protein (GFP) and an erythromycin resistance (Erm^r^)-encoding cassette at their Tn*7* sites (9).

### Genetic manipulation of V. fischeri strain ES114.

Mutant derivatives of V. fischeri strain ES114 were generated using *tfoX*-mediated transformation (38) and PCR and splicing by overhang extension (PCR-SOE) products derived with recently described methodology (39). Briefly, sequences (∼500 bp) upstream and downstream of the target gene were amplified by PCR with high-fidelity polymerase KOD using strain ES114 DNA as the template. These flanking sequences were then fused using PCR SOEing with an internal antibiotic resistance cassette (trimethoprim resistance for single mutants or spectinomycin resistance for double mutants) generated with primers 2089 and 2090 and template pMLC2 or pKV520, respectively. Specifically, flanking sequences were obtained with the following primer sets (Table S2): Δ*VF_A0023*, primers 2400 and 2401 and primers 2402 and 2403; Δ*VF_A0024*, primers 2404 and 2405 and primers 2406 and 2407, and Δ*VF_A0023-24* double deletion, primers 2403 and 2761 and primers 2406 and 2407. Complementation cassettes were obtained by a similar approach to insert the target genes into a region between *yeiR* and *glmS* as described previously (39) using flanking primers 2290 and 2090 with template pKV502, primers 2196 and 1487 with template pKV503, and specific primers as follows: primers 2464 and 2465 for *VF_A0023* and primers 2438 and 2439 for *VF_A0024*.

### Colonization assay.

V. fischeri strains were grown at 28°C overnight in Luria-Bertani salt (LBS) media (40), supplemented with 5 μg/ml erythromycin for the labeled strains carrying Erm^r^. The overnight cultures were diluted 100× in seawater tryptone (SWT) liquid medium and grown until mid-exponential phase at 28°C. Bacteria were diluted to a final concentration aimed at providing an inoculum of ∼5,000 CFU/ml in 100 ml of filter-sterilized ocean water (FSOW), into which newly hatched juvenile *E. scolopes* squids were placed.

The outcomes of single or cocolonization experiments were determined as follows: after a 3-h exposure to the inoculum, each juvenile squid was transferred to a vial containing 4 ml FSOW and incubated overnight in a room with a 12-h/12-h day/night cycle. At 24 h postinoculation, the squids were frozen at –80°C in 700 μl of FSOW, thawed, and individually homogenized. Dilutions of the homogenate were spread on LBS agar medium, and the number of CFU per light organ was calculated. When needed, a fluorescence dissecting scope was used to count colonies of GFP-labeled bacteria.

To determine whether a 30-min inoculation was sufficient to initiate a successful squid colonization, after that exposure, the squids were carefully rinsed in individual vials containing 4 ml FSOW. The rinsing procedure consisted of transferring each squid between vials of FSOW after 1 min (twice) and 5 min (once). The animals were then placed into a vial of FSOW and incubated overnight. At 24 h postinoculation, the bioluminescence of each squid was determined using a luminometer (Turner Designs, Inc.). The presence of luminescence was confirmed as a valid indication of a successful colonization.

### Genome sequences.

Strains with previously determined genome sequences are indicated in Table S3. For the strains isolated from C. multispinulosus, E. morsei, E. tasmanica, and L. pealei, genomic DNA was extracted using the protocol of the Qiagen genomic DNA buffer set with Qiagen genomic tip 100G (Qiagen Inc., Valencia, CA, USA). Sequencing libraries were prepared using the Nextera DNA Library Preparation kit (Illumina). Each strain was barcoded and sequenced in a 100-bp x 100-bp multiplex run on the HiSeq 2000 (Illumina) at the MIT BioMicroCenter (Cambridge, MA). After importing the reads into CLC Genomics Workbench (version 8.0.2), we trimmed adapter sequences and low-quality regions using the following parameters and settings: ambiguous limit, 0 bp; quality limit,  0.005; maximum number of nucleotides in reads, 100 bp; minimum number of nucleotides in reads, 90 bp. Overlapping paired reads were then merged, and the final assemblies were performed using the CLC assembler with read-mapping correction using the following parameters and settings: minimum contig length, 200 bp; mismatch cost, 2; insertion cost, 3; deletion cost, 3; length fraction, 0.5; similarity fraction, 0.8. Open reading frames (ORFs) were identified using Prodigal (version 2.6.3) (41), and the functions for ORFs were inferred using the svr_assign_using_figfams script in the SEED server suite of tools (www.theseed.org).

10.1128/mBio.03407-19.3 TABLE S3 Genomic information for V. fischeri strains. Download Table S3, PDF file, 0.03 MB. Copyright © 2020 Bongrand et al. 2020 Bongrand et al. This content is distributed under the terms of the Creative Commons Attribution 4.0 International license.

### Genome analysis.

The OrthoMCL software (30) was used to determine clusters of orthologous proteins across the 44 tested V. fischeri genomes (Table S1). After filtering out protein sequences that were either less than 10 amino acids in length or over 20% of stop codons, 174,175 sequences were included for the analysis. A cutoff E value of 1 × 10^−5^ was used for the all-versus-all BLASTp (42). The 1,784 single-copy clusters that were obtained were individually aligned at the protein level with mafft-linsi (43), then back-translated with backtranseq (44), realigned at the nucleotide level with mafft-FFT-NS-I (43), trimmed with trimAl (45) for sites with over 50% gaps, and concatenated for tree construction. Phylogenetic trees were constructed with RAxML (46) using the GTRGAMMA model with 1,000-bootstrap pseudoreplications.

### Data availability.

This Whole Genome Shotgun project has been deposited at DDBJ/ENA/GenBank under the accession numbers listed in Table S3 and at https://www.ncbi.nlm.nih.gov/genome/genomes/724?.
