# Supplementary material for: Using Colonization Assays and Comparative Genomics To Discover Symbiosis Behaviors and Factors in Vibrio fischeri
Source: mBio. 2020 Mar 3;11(2):e03407-19. doi: 10.1128/mBio.03407-19 (PMC7064787; doi:10.1128/mBio.03407-19)
Supplement: TABLE S1 [file mBio.03407-19-st001.pdf]

Supplementary Table S1. Strains described in this study.

Strain name	Alias	Host species*	Year isolated	Geographic location	Reference or source
CM1.1	*cmult*.1.1	*C. multispinulosis*	2006	Saga Market, Kochi, Shikoku, Japan	(1)
CM1.2	*cmult*.1.2	*C. multispinulosis*	2006	Saga Market, Kochi, Shikoku, Japan	(1)
EM1.1	*emors*.1.1	*E. morsei*	1995	Tokyo Bay, Honshu, Japan	(2)
EM1.2	*emors*.1.2	*E. morsei*	1995	Tokyo Bay, Honshu, Japan	P. Dunlap
EM2.1	*emors*.2.1	*E. morsei*	1995	Tokyo Bay, Honshu, Japan	P. Dunlap
EM2.2	*emors*.2.2	*E. morsei*	1995	Tokyo Bay, Honshu, Japan	P. Dunlap
EM3.1	*emors*.3.1	*E. morsei*	1995	Tokyo Bay, Honshu, Japan	P. Dunlap
EM3.2	*emors*.3.2	*E. morsei*	1995	Tokyo Bay, Honshu, Japan	P. Dunlap
EM4.1	*emors*.4.1	*E. morsei*	1995	Tokyo Bay, Honshu, Japan	P. Dunlap
EM4.2	*emors*.4.2	*E. morsei*	1995	Tokyo Bay, Honshu, Japan	P. Dunlap
EM5.1o	*emors*.5.1o	*E. morsei*	1995	Tokyo Bay, Honshu, Japan	P. Dunlap
EM5.1t	*emors*.5.1t	*E. morsei*	1995	Tokyo Bay, Honshu, Japan	P. Dunlap
EM5.2	*emors*.5.2	*E. morsei*	1995	Ago Bay, Honshu, Japan	P. Dunlap
EM6.1	*emors*.6.1	*E. morsei*	1995	Ago Bay, Honshu, Japan	(2)
EM6.2	*emors*.6.2	*E. morsei*	1995	Ago Bay, Honshu, Japan	(2)
EM7.1	*emors*.7.1	*E. morsei*	1995	Ago Bay, Honshu, Japan	(2)
EM7.2	*emors*.7.2	*E. morsei*	1995	Ago Bay, Honshu, Japan	(2)
EM8.2	*emors*.8.2	*E. morsei*	1995	Ago Bay, Honshu, Japan	(2)
EM8.7	*emors*.8.7	*E. morsei*	1995	Ago Bay, Honshu, Japan	(2)
EM9.1	*emors*.9.1	*E. morsei*	1995	Ago Bay, Honshu, Japan	(2)
EM9.2	*emors*.9.2	*E. morsei*	1995	Ago Bay, Honshu, Japan	(2)
EM10.1	*emors*.10.1	*E. morsei*	1995	Ago Bay, Honshu, Japan	(2)
EM10.3	*emors*.10.3	*E. morsei*	1995	Ago Bay, Honshu, Japan	(2)
EM11.1	*emors*.11.1	*E. morsei*	1995	Ago Bay, Honshu, Japan	(2)
EM11.2	*emors*.11.2	*E. morsei*	1995	Ago Bay, Honshu, Japan	(2)
ES114		*E. scolopes*	1988	Kaneohe Bay, Oahu, HI, USA	(3)
ES213		*E. scolopes*	1990	Maunalua Bay, Oahu, HI, USA	(4)
KB1A98		*E. scolopes*	2005	Kaneohe Bay, Oahu, HI, USA	(5)
KB2B1		*E. scolopes*	2005	Kaneohe Bay, Oahu, HI, USA	(5)
KB4B5		*E. scolopes*	2005	Kaneohe Bay, Oahu, HI, USA	(5)
MB11B1		*E. scolopes*	2005	Maunalua Bay, Oahu, HI, USA	(5)
MB13B1		*E. scolopes*	2005	Maunalua Bay, Oahu, HI, USA	(5)
MB13B2		*E. scolopes*	2005	Maunalua Bay, Oahu, HI, USA	(5)
MB13B3		*E. scolopes*	2005	Maunalua Bay, Oahu, HI, USA	(5)
MB14A3		*E. scolopes*	2005	Maunalua Bay, Oahu, HI, USA	(5)
MB15A4		*E. scolopes*	2005	Maunalua Bay, Oahu, HI, USA	(5)
MB15A5		*E. scolopes*	2005	Maunalua Bay, Oahu, HI, USA	(5)
VLS2		*E. scolopes*	1990	Kaneohe Bay, Oahu, HI, USA	(6)
ET1.1	*etasm.1.1*	*E. tasmanica*	2000	Launceston, Tasmania	(7)
ET2.1	*etasm*.2.1	*E. tasmanica*	2000	Launceston, Tasmania	(7)
LP1.1	*lpeal*.1.1	*L. pealei*	1989	Woods Hole, MA	(7)
LP1.2	*lpeal*.1.2	*L. pealei*	1989	Woods Hole, MA	P. Dunlap
MJ11		*M. japonica*	1994	Japan	(8)
SR5		*S. robusta*	1995	Banyuls sur Mer, France	(9)

**C.: Coelorinchus; E.: Euprymna; L.: Loligo; M.: Monocentris; S.: Sepiola*

All are squid symbionts except isolates from *C. multispinulosis* and *M. japonica*

All strains have been isolated from light organs except *lpeal.1.1 and lpeal1.2,* which come from an accessory nidamental gland

Strains numbered with the same first digit come from the same specimen; *i.e*., EM1.1 and EM1.2, or MB15A4 and MB15A5.

1. Dunlap P V., Ast JC, Kimura S, Fukui A, Yoshino T, Endo H. 2007. Phylogenetic analysis of host-symbiont specificity and codivergence in bioluminescent symbioses. Cladistics 23:507–532.
2. Ast JC, Urbanczyk H, Dunlap P V. 2009. Multi-gene analysis reveals previously unrecognized phylogenetic diversity in *Aliivibrio*. Syst Appl Microbiol 32:379–386.
3. Boettcher KJ, Ruby EG. 1990. Depressed light emission by symbiotic *Vibrio fischeri* of the sepiolid squid *Euprymna scolopes.* J Bacteriol 172:3701–3706.
4. Boettcher KJ, Ruby EG. 1994. Occurrence of plasmid DNA in the sepiolid squid symbiont *Vibrio fischeri*. Curr Microbiol 29:279–286.
5. Wollenberg MS, Ruby EG. 2009. Population structure of *Vibrio fischeri* within the light organs of *Euprymna scolopes* squid from two Oahu (Hawaii) populations. Appl Environ Microbiol 75:193–202.
6. Lee KH, Ruby EG. 1994. Effect of the squid host on the abundance and distribution of symbiotic *Vibrio fischeri* in nature. Appl Environ Microbiol 60:1565–1571.
7. Urbanczyk H, Ast JC, Higgins MJ, Carson J, Dunlap P V. 2007. Reclassification of *Vibrio fischeri*, *Vibrio logei*, *Vibrio salmonicida* and *Vibrio wodanis* as *Aliivibrio fischeri* gen. nov., comb. nov., *Aliivibrio logei* comb. nov., *Aliivibrio salmonicida* comb. nov. and *Aliivibrio wodanis* comb. nov. Int J Syst Evol Microbiol 57:2823–2829.
8. Mandel MJ, Stabb E V., Ruby EG. 2008. Comparative genomics-based investigation of resequencing targets in *Vibrio fischeri*: Focus on point miscalls and artefactual expansions. BMC Genomics 9:138.
9. Fidopiastis PM, Von Boletzky S, Ruby EG. 1998. A new niche for *Vibrio logei,* the predominant light organ symbiont of squids in the genus *Sepiola*. J Bacteriol 180:59–64.
