# Supplementary material for: Using Colonization Assays and Comparative Genomics To Discover Symbiosis Behaviors and Factors in Vibrio fischeri
Source: mBio. 2020 Mar 3;11(2):e03407-19. doi: 10.1128/mBio.03407-19 (PMC7064787; doi:10.1128/mBio.03407-19)
Supplement: TABLE S2 [file mBio.03407-19-st002.pdf]

Supplementary Table S2. Primers used in this study.

Primer	Sequence[1]
1487	GGTCGTGGGGAGTTTTATCC
2089	CCATACTTAGTGCGGCCGCCTA
2090	CCATGGCCTTCTAGGCCTATCC
2196	TCCATACTTAGTGCGGCCGCCTA
2290	AAGAAACCGATACCGTTTACG
2400	CTAATCTCAGCTCGCACTTC
2401	taggcggccgcactaagtatggGATAGCTACTGTTGCGATGC
2402	ggataggcctagaaggccatggGATACAGCCGCTGAACTATC
2403	GGGAAAGAGTCAAATTATACCG
2404	CCACACCATCAACTAAAGCAAC
2405	taggcggccgcactaagtatggAAAAGAGCCAAGTTCAGTGACC
2406	ggataggcctagaaggccatggTACGGTGGTACGAAGTAGTTTG
2407	GAGCTTCCTCTTGAAAGAACG
2438	ggataggcctagaaggccatggGATAGCTACTGTTGCGATGC
2439	taggcggccgcactaagtatggAACTACTTCGTACCACCGTAC
2464	ggataggcctagaaggccatggAAAAGAGCCAAGTTCAGTGACC
2465	taggcggccgcactaagtatggaCCAATACGACGGCTAATAATGG
2761	taggcggccgcactaagtatggGATACAGCCGCTGAACTATC

[1]Lower-case sequences represent non-native overlapping sequences used to generate a fused PCR product
