# Supplementary material for: Using Colonization Assays and Comparative Genomics To Discover Symbiosis Behaviors and Factors in Vibrio fischeri
Source: mBio. 2020 Mar 3;11(2):e03407-19. doi: 10.1128/mBio.03407-19 (PMC7064787; doi:10.1128/mBio.03407-19)
Supplement: TABLE S3 [file mBio.03407-19-st003.pdf]

Supplementary Table S3. Genomic information for *V. fischeri* strains.

Strain name	RefSeq assembly accession number	Genome size (Mb)	Number of proteins	GC content
CM1.1	GCA_009728125.1	3.6	3703	38.7
CM1.2	GCA_009728075.1	3.6	3702	38.7
EM1.1	GCA_009728065.1	3.8	3811	38.7
EM1.2	GCA_009728085.1	3.8	3766	39.0
EM2.1	GCA_009727955.1	3.8	3758	39.0
EM2.2	GCA_009727925.1	3.8	3773	39.0
EM3.1	GCA_009727945.1	3.8	3797	39.0
EM3.2	GCA_009727905.1	3.8	3828	39.0
EM4.1	GCA_009727825.1	4.0	4141	38.9
EM4.2	GCA_009727845.1	3.7	3745	38.9
EM5.1o	GCA_009727875.1	3.7	3781	39.0
EM5.1t	GCA_009727835.1	3.7	3776	39.0
EM5.2	GCA_009727805.1	3.8	3926	39.0
EM6.1	GCA_009727735.1	3.7	3773	39.0
EM6.2	GCA_009727745.1	3.7	3771	39.0
EM7.1	GCA_009727755.1	3.8	3833	39.0
EM7.2	GCA_009727725.1	3.7	3758	39.0
EM8.2	GCA_009727705.1	3.9	4040	38.8
EM8.7	GCA_009727685.1	3.8	3843	39.0
EM9.1	GCA_009727605.1	4.0	4050	38.8
EM9.2	GCA_009727645.1	3.8	3868	39.0
EM10.1	GCA_009728045.1	3.8	3874	38.9
EM10.3	GCA_009728025.1	3.8	3897	38.9
EM11.1	GCA_009727995.1	3.9	4014	38.9
EM11.2	GCA_009727975.1	3.7	3700	39.0
ES114	GCA_000011805.1	4.3	3814	38.4
ES213	GCA_001640315.1	4.4	3962	38.2
KB1A98	GCA_001640325.1	4.2	3689	38.2
KB2B1	GCA_001640305.1	4.4	3896	38.2
KB4B5	GCA_001640505.1	4.2	3677	38.2
MB11B1	GCA_001640385.1	4.5	4082	38.0
MB13B1	GCA_001640415.1	4.3	3910	38.1
MB13B2	GCA_001640445.1	4.4	3899	38.2
MB13B3	GCA_001640465.1	4.5	4086	38.0
MB14A3	GCA_001640375.1	4.1	3676	38.2
MB15A4	GCA_001640455.1	4.2	3763	38.2
MB15A5	GCA_001640525.1	4.1	3615	38.2
VLS2	GCA_001640545.1	4.1	3642	38.3
ET1.1	GCA_009727625.1	3.7	3789	38.9
ET2.1	GCA_009727615.1	3.7	3768	39.0
LP1.1	GCA_009727585.1	3.8	3845	39.1
LP1.2	GCA_009727525.1	3.8	3847	39.1
MJ11	GCA_000020845.1	4.5	3948	38.2
SR5	GCA_000241785.1	4.3	3754	38.4
